# Case Report: Transient antenatal bartter syndrome in an extremely preterm infant with a novel *MAGED2* variant

**DOI:** 10.3389/fped.2022.1093268

**Published:** 2023-02-02

**Authors:** Hongyuan Yang, Zhiyong Liu, Yaying Wu, Jinglin Xu, Ying He, Ruiquan Wang, Weifeng Zhang, Dongmei Chen

**Affiliations:** ^1^ Department of Neonatology, Quanzhou Maternity and Children's Hospital, Quanzhou, China; ^2^ Department of Plastic Surgery, Quanzhou Maternity and Children's Hospital, Quanzhou, China

**Keywords:** *MAGED2*, antenatal bartter's syndrome, extremely preterm infant, transient haematuria, acute kidney injury

## Abstract

Variants in the *MAGED2* may cause antenatal transient Bartter syndrome, which is characterised by polyhydramnios, preterm labour, postnatal polyuria, hypokalaemia and metabolic alkalosis. Transient gross hematuria and acute kidney injury in such cases have not been reported previously. The patient, a boy, was born at a gestational age of 27 + 5 weeks. Polyhydramnios has been detected at 24 weeks of gestation. Polyuria, hyponatraemia, hypokalaemia, weight loss, transient hematuria and acute kidney injury occur after birth. The urinary ultrasonography showed no abnormality, and after a month of treatment with liquid electrolytes and nutritional management, the clinical symptoms improved. Whole-exome sequencing revealed a variant in *MAGED2*: c.1426C > T, p.Arg476X, inherited from the mother, who was healthy. During the 1-year follow-up, the child grew and developed with normal renal function and electrolyte levels. This is the first report of transient antenatal Bartter syndrome caused by a *MAGED2* variant in China in an extremely preterm infant who exhibited previously unreported symptoms: transient hematuria and acute kidney injury. This newly found variant expands the spectrum of genetic variants associated with antenatal Bartter syndrome; it can be detected by early genetic testing and overmedication, thereby avoided.

## Introduction

Bartter syndrome is a group of rare inherited renal tubular diseases resulting from a disorder of salt reabsorption in the thick ascending limb of Henle's loop (1). There exist two main types of Bartter syndrome: antenatal Bartter syndrome (aBS) and classic Bartter syndrome. The first is characterised by polyhydramnios and preterm birth as results of severe fetal polyuria, but these clinical manifestations disappear completely in the first few months of life (2).

In 2016, variants in *MAGED2* in 13 male infants from seven different families who had transient aBS were reported by Laghmani et al. (3), who identified *MAGED2* as a causative gene for aBS. The *MAGED2*, located at Xp11.2, encodes a protein that gradually translocates from the cytoplasm to the nucleoplasm after interphase and upon nucleolar stress and is therefore considered to play a role in cell cycle regulation. This gene is linked to several types of cancer, including breast cancer and melanoma.

Patients with aBS have severe symptoms. Long-term treatment with nonsteroidal anti-inflammatory drugs was previously considered necessary, but recent studies have demonstrated good short-term and long-term prognoses without medication. However, aBS can lead to extremely preterm birth, which may be lethal.

We report a case of transient aBS caused by a novel *MAGED2* variant, in which the infant exhibited polyuria, hematuria and renal impairment soon after birth but had a favourable long-term outcome. This is the first recorded case of an extremely preterm infant with aBS in the Chinese population.

## Case report

**Figure 1:**
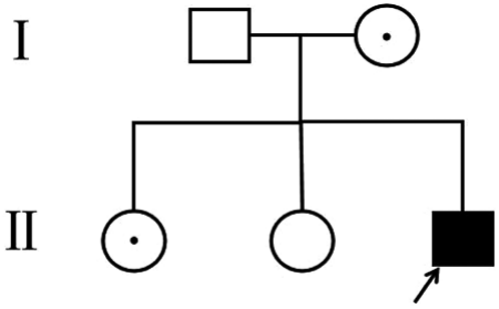
Pedigree of the family. The arrow denotes the proband and the hollow symbols represent the unaffected members and add a little to the hollow symbols represent the carrier.

**Figure 2:**
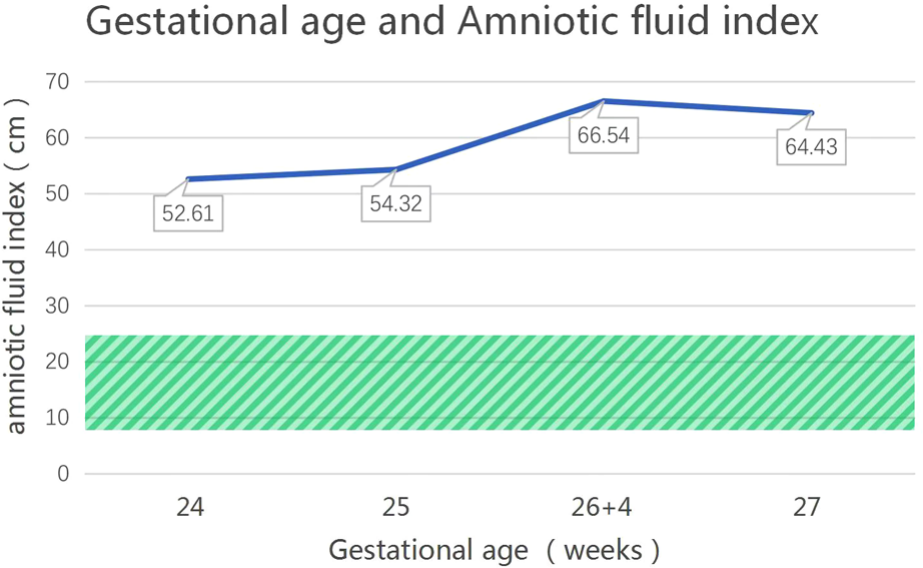
The changes in the amniotic fluid index during pregnancy for the patient with an *MAGED2* variant during pregnancy. (The normal range of amniotic fluid index is 8–25 cm, as shown in the shaded area).

The patient, a boy, was born to a family in Quanzhou, a city in South China (Figure 1). His father was 40 years old, and his mother was 36 years old. Both were healthy and had no family history of hereditary diseases. His mother's prenatal examination is as follows: blood pressure (118/73 mmHg), sodium (137 mmol/L), chloride (100.9 mmol/L), potassium (3.6 mmol/L), Urea nitrogen (1.49 mmol/L) and Creatinine (30.3 μmol/L). This child resulted from the mother's third natural conception, and a non-invasive DNA test at 15 weeks of gestation showed no abnormality. Polyhydramnios was detected at 24 weeks of gestation (Figure 2). Ultrasonography revealed multiple spot-like strong echoes in the left ventricle and enhanced echoes in part of the both intestine. Karyotyping and *Chromosomal Microarray Analysis* examination by amniocentesis, performed at 25 weeks of gestation, revealed no abnormality. At 26 weeks, the mother developed chest tightness, abdominal distension and irregular uterine contractions and was prescribed dexamethasone 6 mg four times during the course of pregnancy and magnesium sulphate 1500 mg per day for 3 days. At 27 + 3 weeks, natural delivery was unavoidable; the infant weighed 1350 g (Apgar scores: 8–9–9), and approximately 2100 ml of clear amniotic fluid was delivered. After birth, the infant was transferred to the neonatal intensive care unit of a tertiary hospital, where he received invasive mechanical ventilation, pulmonary surfactant, antibiotics and intravenous nutrition.

The infant's renal function was normal 1 h after birth, but he had polyuria (5.0 ml/kg per hour on average) during the first 3 days, and gross hematuria developed 2 days after birth but disappeared 4 days later. His body weight decreased by 30.4% to 940 g in the first 6 days after birth. Electrolyte analysis revealed low levels of sodium (131 mmol/L), chloride (95 mmol/L) and potassium (3.32 mmol/L) but normal levels of magnesium. The infant was given sodium, chloride and potassium supplements and electrolyte fluids intravenously. Electrolyte levels returned to normal on day 7, renal function returned to normal on day 14 (Table 1), and birth weight was regained on day 20. The infant was receiving full-time enteral nutrition (breastfed on demand) 48 days after birth, with an additional oral 10% potassium chloride solution (1 ml, three times a day). He was discharged in good condition 83 days after birth, which corresponded to 39 + 1 weeks of corrected gestational age. After being discharged from the hospital, he was regularly given 10% potassium chloride oral solution, 1 ml at a time, three times a day, for 3 months (corrected age of 2 months 24 days). All the examinations during hospitalisation, including colour Doppler ultrasonography of the urinary system, colour ultrasonography of the heart and the head, measurement of auditory brainstem evoked potentials and blood tandem mass spectrometry, revealed no obvious abnormality. His renal function was normal, with potassium levels fluctuating between 3.5 and 4.1 mmol/L. He exhibited good physical, athletic and intellectual development 12 months after birth (corrected age of 9 months).

**Table 1:** Changes in renal function–related indexes after birth.

Age	1 day	4 days	6 days	9 days	14 days	1 month	3 months	6 months	1 year
Urea nitrogen (mmol/L)	2.21	7.15	37.05	11.32	7.34	2.02	2.33	5.42	3.81
Creatinine (μmol/L)	33.3	45.2	115.5	39.8	15.1	18.8	12.5	16.9	14.5

## Genetic analysis

**Figure 3:**
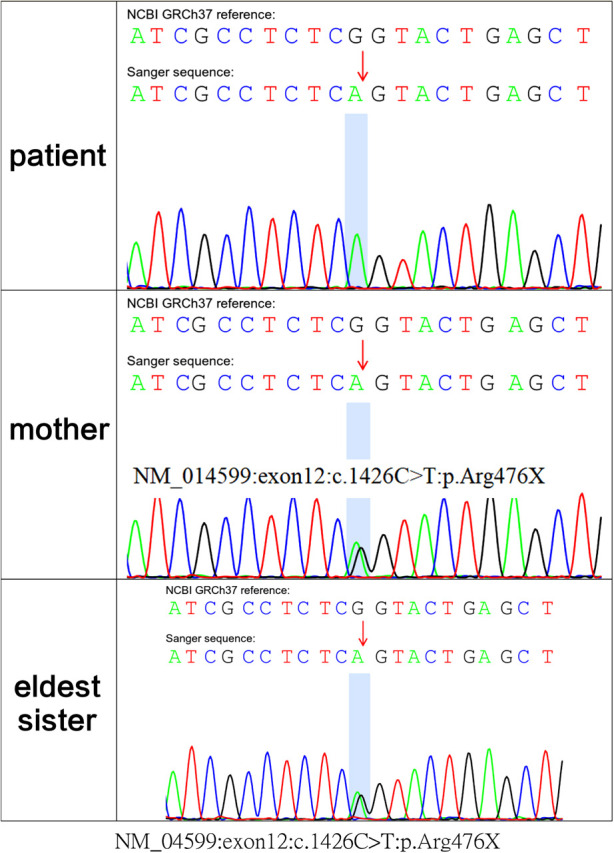
whole-exome sequencing showed c.1426C > T:p.Arg476X of the MAGED2 (NM_014599) variant in the fetus; his mother and eldest sister carried the variant in the heterozygous state.

Because polyuria, hyponatraemia, hypokalaemia, hematuria and acute kidney injury had occurred early in life, whole-exome sequencing was performed, with parental consent, 10 days after the patient's birth. Sequencing results, received 30 days after his birth, indicated a hemizygous variant in MEGED2 (c.1426C > T:p.Arg476X) on the X chromosome; the variant was thus inherited from his mother (Figure 3). The variant was considered potentially pathogenic, as suggested by the American College of Medical Genetics and Genomics grading (PVS1 (strong), PM2 (supporting) and PP4). The diagnosis was, therefore, transient aBS caused by a *MAGED2* variant, according to the clinical manifestations and genetic analysis results. Sanger sequencing confirmed that both sisters of the patient were carriers of the variant.

## Discussion

Bartter syndrome is a group of inherited renal tubular diseases characterised by secondary aldosteronism, hypokalaemia and hypochloraemic metabolic alkalosis; blood pressure is not affected (4). The *MAGED2* encodes melanoma-associated antigen D2 (MAGE-D2), required for activation of the cyclic adenosine monophosphate/protein kinase A pathway under hypoxic conditions (5). MAGE-D2 participates in the biofunction of Na + -K + -2Cl− cotransporters (NKCC2) and Na + -2Cl− cotransporters (NCC), possibly by cooperating with Hsp40 and Gs*α* proteins, respectively. MAGE-D2 is, therefore, essential for fetal renal salt absorption, amniotic fluid homeostasis and the maintenance of normal pregnancy (6). The *MAGED2* variant identified in our case, c.1426C > T:p.Arg476X, is a novel nonsense variant. Among the aBS cases reported in recent years, a total of 31 variants in the *MAGED2* have been reported: 6 nonsense variants, 8 frameshift variants, 7 splicing variants, 6 deletions and 4 missense variants. The relationship between genotype and phenotype has not yet been fully elucidated. Identification of more genotypes may help reveal hotspot variants and facilitate genetic diagnosis in pregnant women with polyhydramnios.

Wu et al. (7) reported a case of polyhydramnios 21 weeks before delivery and identified a new frameshift variant in the *MAGED2* (NM_177433.1) through amniocentesis. Two amniotic fluid reductions were performed at 24 weeks and 29 + 5 weeks. The infant was delivered at 35 weeks without polyuria or other abnormalities. Legrand et al. (8) showed that in all pregnant women in their study whose fetuses had the *MAGED2* variant, severe polyhydramnios occurred at 18–27 weeks of gestation; in such cases, amniotic fluid reduction is recommended. Almost all the infants were born prematurely; 38% of the fetuses were born at <28 weeks, all of whom had more severe clinical manifestations. In the case of our patient, polyhydramnios developed at 24 weeks of gestational age, but no amniotic fluid reduction was performed. The severe increase in amniotic fluid is probably a key reason for extremely preterm birth. Because of the high mortality rate among children born extremely preterm and the aggravation of clinical manifestations after birth, amniotic fluid reduction may be an appropriate option for fetuses with aBS that have severe antenatal polyhydramnios.

The clinical manifestations in our patient were similar to those in previous cases of transient Bartter syndrome caused by *MAGED2* variants. However, the patient developed transient hematuria and acute kidney injury early in the course of the disease, which were not reported in the past. Acute kidney injury was accompanied by severe weight loss, which may have been related to dehydration. After fluid replacement therapy, renal function improved, which suggests that early identification of aBS and active fluid intervention are critical for extremely preterm infants with aBS. Transient hematuria occurs even earlier than acute kidney injury and may be associated with excessive fluid loss during preterm labour, but the exact cause is unclear. Our case suggests that hematuria and acute kidney injury may also be considered manifestations of aBS, which may enable early diagnosis.

The *MAGED2* variant is one of the causes of X-linked recessively inherited transient aBS. Our patient is the first reported extremely premature infant with transient aBS caused by *MAGED2* variant in China. Although transient hematuria and acute kidney injury developed after birth, the prognosis at the 1-year follow-up visit was good. For children with antenatal polyhydramnios, polyuria, electrolyte imbalance, hematuria and acute kidney injury after birth, genetic screening should be performed in addition to colour Doppler ultrasonography of the urinary system so that excessive intervention, especially invasive operations such as renal puncture, can be avoided.

## Data Availability

The original contributions presented in the study are included in the article/Supplementary Material, further inquiries can be directed to the corresponding author/s.
